# The Effects of Resveratrol in Rats with Simultaneous Type 2 Diabetes and Renal Hypertension: a Study of Antihypertensive Mechanisms

**Published:** 2015-03

**Authors:** Masoud Mozafari, Ali Akbar Nekooeian, Mohammad Reza Panjeshahin, Hamid Reza Zare

**Affiliations:** 1 Cardiovascular Pharmacology Research Lab, Department of Pharmacology, School of Medicine, Shiraz University of Medical Sciences, Shiraz, Iran;; 2 Department of Pharmacology, School of Medicine, Shiraz University of Medical Sciences, Shiraz, Iran;; 3 Department of Immunology, School of Medicine, Shiraz University of Medical Sciences, Shiraz, Iran

**Keywords:** Resveratrol, Diabetes mellitus, Renal hypertension, Oxidative stress, Nitric oxide, Rat

## Abstract

**Background:**

Resveratrol has beneficial effects on cardiovascular system. This study aimed at examining antidiabetic and antihypertensive effects of resveratrol in rats with simultaneous type 2 diabetes and renal hypertension.

**Methods:**

Eight groups (8-10 each) of male Spargue-Dawley rats, including a control, a diabetic (induced by streptozotocin and nicotinamide), a renal hypertensive (induced by placing plexiglas clips on the left renal arteries), a sham, a simultaneously hypertensive-diabetic receiving vehicle, and 3 simultaneous hypertensive-diabetic receiving resveratrol at 5, 10 or 20 mg/kg/day were used. Four weeks after the induction of diabetes, renal hypertension was induced and animals were given vehicle or resveratrol for the next four weeks. Afterwards, blood pressure and glucose, serum markers of oxidative stress were measured and animal’s aortic rings were used for isolated studies.

**Results:**

Serum malondialdehyde, systolic blood pressure, heart rate, fasting blood glucose, maximal response and effective concentration 50 of phenylephrine, and inhibitory concentration 50 of acetylcholine of hypertensive-diabetic group receiving vehicle were significantly higher than those of the control group, and treatment with resveratrol caused significant reduction of these variables. Moreover, serum superoxide dismutase, glutathione reductase, and maximal response to acetylcholine of hypertensive-diabetic group receiving vehicle were significantly lower than those of the control group, and treatment with resveratrol caused significant increase of these variables.

**Conclusion:**

The findings indicate that resveratrol has antidiabetic and antihypertensive effects, which may be partly due to antioxidant mechanism. They also show that antihypertensive effect of resveratrol may be additionally mediated by improving the release of nitric oxide and sympathoplegic activities.

## Introduction

Resveratrol (3,5,4-trihydroxystilbene ), an important polyphenolic component of red grapes, has been hypothesized to mediate its cardioprotective effects.^1^ Clinical and experimental studies have shown that resveratrol has beneficial cardiovascular effects. It has been shown to reduce blood pressure in spontaneously hypertensive rats,^2^ DOCA-salt hypertensive rats,^3^ two-kidney, one-clip hypertensive rats,^4^ relaxed precontracted vascular segments from spontaneously hypertensive rats,^2^ DOCA-salt hypertensive rats,^3^ and female normotensive guinea-pigs.^5^ The antihypertensive or vasorelaxing effects of resveratrol have been attributed to increased expression of endothelial nitric synthase,^6^ improved nitric oxide (NO) release and endothelium-dependent vasorelaxation,^2^ decreased endothelin-1 and angiotensin II production,^7^ decreased sympathetic activity,^8^ and decreased oxidative stress.^9^

Earlier studies have shown the beneficial effects of resveratrol in experimental and human diabetes. Resveratrol was shown to reduce blood glucose in experimental^10^ and human type 2 diabetes.^11^ Antidiabetic effects of resveratrol have been attributed to preservation of beta cells,^12^ increase of insulin release,^13^ enhancement of insulin sensitivity,^14^ and decrease of oxidative stress.^15^

Hypertension and diabetes mellitus are increasingly occurring together in human, and when occur concurrently; they make patients more vulnerable to other cardiovascular disease and death.^16^ Although the effects of resveratrol have been investigated in experimental hypertension and diabetes separately, its effects have rarely been investigated in a model simultaneous hypertension and diabetes. The objective of this study was to examine the antidiabetic and antihypertensive effects of resveratrol in a rat model of simultaneous type 2 diabetes mellitus and renal hypertension.

## Materials and Methods

*Animals*

Male Sprague-Dawley rats (n=80), weighting 200-250 g, were obtained from Laboratory Animal Breeding Centre, Shiraz University of Medical Sciences (Shiraz, Iran) and kept under standard conditions (12 h light⁄dark cycle, 25-35% humidity, 20-22°C temperature) with standard diet and water ad libitum. All procedures were approved by the University Committee for Care and Use of Animals.

*Induction of Type 2 Diabetes and Renal Hypertension*

Rats were injected intraperitoneally with 110 mg/kg nicotinamide (NA) (Sigma-Aldrich Chemical Co. (Steinheim, Germany) and 65 mg/kg streptozotocin (STZ) (Teva Parenteral Medicine Inc., Irvine, CA, USA) or distilled water as vehicle (to constitute the control group). Seven days later, animals’ blood glucose levels were determined using a glucometer (Accu-check® active, Germany), and those with a fasting blood glucose higher than 126 mg/dl were considered as having type 2 diabetes.^17^

Four weeks after the induction of diabetes, control and type 2 diabetic animals were subjected to sham-operation or induction of two-kidney, one clip renal hypertension by placing self-made solid plexiglass clips on the left renal arteries as previously described.^18^ Briefly, animals were anesthetized with 60 mg/kg ketamine (Pan Pharam, Trittau, Schleswig Holstein, Germany) and 8 mg/kg Xylazine (Alfasan, Woerden, The Netherland), and through a left flank incision the left renal arteries were exposed and dissected away from renal veins and surrounding tissues. Afterwards, plexiglass clips (internal diameter of 0.20-0.22 mm) were placed on the arteries. Penicillin powder (Jaber Ebne Hayyan, Tehran, Iran) was applied to the incision sites and abdominal wall and skin were sutured using absorbable (catgut) and non-absorbable (silk) suture materials, respectively. Sham-operated animals were subjected to a similar procedure, but no clip was placed around renal arteries. The animals were then recovered from anesthesia and kept in cages of two rats each for 4 weeks under standard condition.

Starting from the day after the operations, 8 groups of animals emerged including: a diabetic control group assigned to receive vehicle (DMC-V), a sham group for renal hypertensive rats assigned to receive vehicle (Sham-V), a renal hypertensive group assigned to receive vehicle (HTN-V), a type 2 diabetic group assigned to receive vehicle (DM-V), a simultaneous type 2 diabetes and renal hypertensive group assigned to receive vehicle (DM+HTN-V), and 3 simultaneous type 2 diabetes and renal hypertensive groups assigned to received resveratrol (R) (Serva Co, Oklahoma, USA) at 5 mg/kg/day (DM+HTN-R5), 10 mg/kg/day (DM+HTN-R10), or 20 mg/kg/day (DM+HTN-R20). Vehicle or resveratrol was administered by oral gavage for the next 4 weeks. On day 28, systolic blood pressure (SBP) and heart rate (HR) were measured using non-invasive tail-cuff method (Chart 5.0 software, PowerLab 4/30, AD Instruments Inc., MA, Australia). Three consecutive blood pressure measurements, which had a difference of less than 5 mmHg, were considered as valid. Those measurements were averaged and their means were taken as values of blood pressure at each time.

After the measurement of SBP and HR, animals were anesthetized with 70 mg/kg thiopental sodium (Biochem GmbH, Vienna, Austria). Fasting blood glucose (FBG) was measured using the glucometer, and blood samples for measurement of serum levels of malondialdehyde (MDA), superoxide dismutase (SOD), glutathione reductase (GR) were collected. The samples were allowed to clot for 30 minutes, centrifuged at 3000 rpm for 20 minutes, and their serums were separated and stored at -80ºC until analysis. The animals were then sacrificed and their thoracic aortas were used for isolated tissue studies. 

*Isolated Aortic Ring Studies*

Isolated thoracic aortas were cleaned from the surrounding connective tissues and cut into 4-5 mm rings. The rings were mounted on hooks connected to force transducers in isolated tissue organ baths (K30, Hugo Sachs Electronik, Germany) filled with 20 ml physiological solution containing the following composition (mmol/L): NaCl 118, KCl 4.7, KH_2_PO_4_ 1.2, CaCl_2_ 2.5, MgSO_4_ 1.2, NaHCO_3_ 25, D-glucose 11.1, bubbled constantly with 95% O_2_ and 5% CO_2_ at a pH of 7.4 and a temperature of 37ºC. Tension was recorded with a four-channel polygraph (model 705/1, Hugo Sachs Electronik, Germany). The tissues were allowed to stabilize for 60 minutes. Then, a full concentration-response to phenylephrine (Phe) (Sigma-Aldrich Chemical Co., Steinheim, Germany) was performed. After two washes and 30 minutes equilibration, the rings were contracted with Phe concentrations that made similar contraction responses (50% of maximal response in the DMC-Veh group) in all groups. Concentration-response curves to acetylcholine (ACh) or sodium nitroprusside (SNP) (both from Sigma-Aldrich Chemical Co., Steinheim, Germany) were performed at the plateau of contractile response to Phe. Concentration-responses to Phe were compared using EC_50_ and maximal response (E_max_). Concentration-responses to ACh or SNP were compared using IC_50_ and E_max_.

*Biochemical Measurements*

Serum level of MDA was determined using MDA ELISA (Bioassay technology laboratory, Shanghai, China) kit. Serum level of SOD and glutathione reductase (GR) was determined using Biorexfars chemical kits (Shiraz, Iran).

*Statistical Analysis*

Data, presented as mean±SEM, were analyzed using One-way Analysis of Variance (ANOVA). Where a significant difference was obtained with one-way ANOVA, the source of the difference was located using Duncan’s Multiple Range test. The data were analyzed using Sigmastat statistical software version 3.0. P value ≤0.05 was considered statistically significant. 

## Results

*Blood Pressure and Heart Rate*

**Figure 1:**
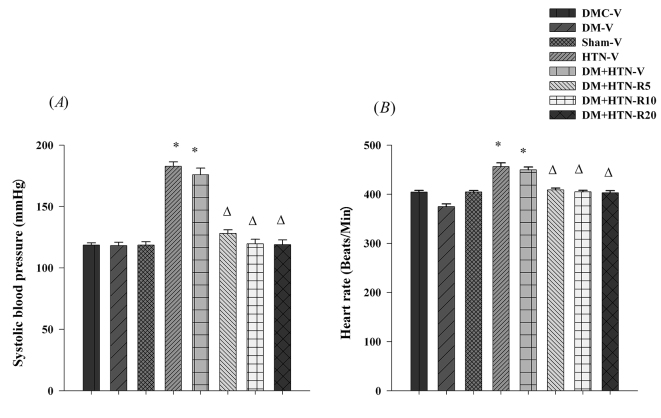
(A) Systolic blood pressure and (B) heart rate for all experimental groups after 4 weeks of treatment with vehicle or resveratrol. DMC-V: Diabetic control group receiving vehicle; Sham-V: Sham-operated receiving vehicle; DM-V: Diabetic receiving vehicle; HTN-V: Hypertensive receiving vehicle; DM+HTN-V: Diabetic hypertensive receiving vehicle; DM+HTN-R5: Diabetic hypertensive receiving 5 mg/kg/day resveratrol; DM+HTN-R10: Diabetic hypertensive receiving 10 mg/kg/day resveratrol; DM+HTN-R20: Diabetic hypertensive receiving 20 mg/kg/day resveratrol; Data are mean±SEM and n=8-10 in each group; *Significant difference (P<0.05) from DMC-V or Sham-V; ∆Significant difference (P<0.05) from DM+HTN-V

There was no significant difference in the SBP of DMC-V, DM-V and Sham-V groups (figure 1A). Systolic blood pressures of HTN-V, DM+HTN-V were significantly higher than those of DM-C-V, Sham-V and DM-V (figure 1A). Systolic blood pressures of resveratrol-treated groups (DM+HTN-R5, DM+HTN-R10 and DM+HTN-R20) were significantly lower than those of the DM+HTN-V group (figure 1A).

The HR of the DM-V group was significantly lower than those of the DMC-V group (figure 1B). The HR of the HTN-V and DM+HTN-V groups were significantly higher than those of the DMC-V, Sham-V and DM-V groups (figure 1B). The HR of DM+HTN-R5, DM+HTN-R10 and DM+HTN-R20 groups were significantly lower than those of the DM+HTN-V group (figure 1B).

*Serum Biochemistry*

Fasting blood glucose of the DM-V and DM+HTN-V groups were significantly higher than those of the DMC-V, Sham-V and HTN-V groups. Fasting blood glucose of DM+HTN-R5, DM+HTN-R10 and DM+HTN-R20 groups were significantly lower than that of the DM+HTN-V group (table 1).

**Table 1:** The values of biochemical markers of experimental groups after 4 weeks of treatment with vehicle or resveratrol

	**FBS ** **(mg/dl)**	**MDA ** **(nmol/ml)**	**SOD ** **(Unit/ml)**	**GR ** **(Unit/L)**
DMC-V	104.9±2.4	0.63±0.07	264.2±39.2	70.6±4.1
DM-V	209.0±4.4*	1.55±0.11*	92.9±11.3*	26.4±2.8*
Sham-V	106.1±2.6	0.64±0.07	243.5±43.9	68.8±3.9
HTN-V	120.4±1.5	1.32±0.13*	147.4±19.7*	39.4±3.1*
DM+HTN-V	199.4±5.8*	1.81±0.45*	116.1±13.8*	30.1±2.4*
DM+HTN-R5	123.6±2.7^Δ^	0.63±0.08^Δ^	265.6±51.1^Δ^	62.8±5.1^Δ^
DM+HTN-R10	106.1±4.2^Δ^	0.64±0.09^Δ^	302.3±62.1^Δ^	64.7±5.1^Δ^
DM+HTN-R20	102.6±5.4^Δ^	0.56±0.07^Δ^	276.1±61.5^Δ^	65.4±3.7^Δ^

DMC-V: Diabetic control group receiving vehicle; Sham-V: Sham-operated receiving vehicle; DM-V: Diabetic receiving vehicle; HTN-V: Hypertensive receiving vehicle; DM+HTN-V: Diabetic hypertensive receiving vehicle; DM+HTN-R5: Diabetic hypertensive receiving 5 mg/kg/day resveratrol; DM+HTN-R10: Diabetic hypertensive receiving 10 mg/kg/day resveratrol; DM+HTN-R20: Diabetic hypertensive receiving 20 mg/kg/day resveratrol; FBS: Fasting blood glucose; MDA: Malondialdehyde; SOD: Superoxide dismutase; GR: Glutathione reductase; Data are mean±SEM and n=8-10 in each group; *Significant difference (P<0.05) with DMC-Veh or Sham-V; ^∆^Significant difference (P<0.05) with DM+HTN-V

Serum levels of MDA of the HTN-V, DM-V and DM+HTN-V groups were significantly higher than those of the DMC-V and Sham-V groups. Serum levels of MDA of DM+HTN-R5, DM+HTN-R10 and DM+HTN-R20 groups were significantly lower than the DM+HTN-V group (table 1).

Serum levels of SOD of the HTN-V, DM-V and DM+HTN-V groups were significantly lower than those of the DMC-V and Sham-V groups. Serum levels of SOD of DM+HTN-R5, DM+HTN-R10 and DM+HTN-R20 groups were significantly higher than the DM+HTN-V group (table 1).

Serum levels of GR of the HTN-V, DM-V and DM+HTN-V groups were significantly lower than those of the DMC-V and Sham-V groups. Serum levels of GR of DM+HTN-R5, DM+HTN-R10 and DM+HTN-R20 groups were significantly higher than the DM+HTN-V group (table 1).

*Isolated Aortic Ring Studies*

**Figure 2:**
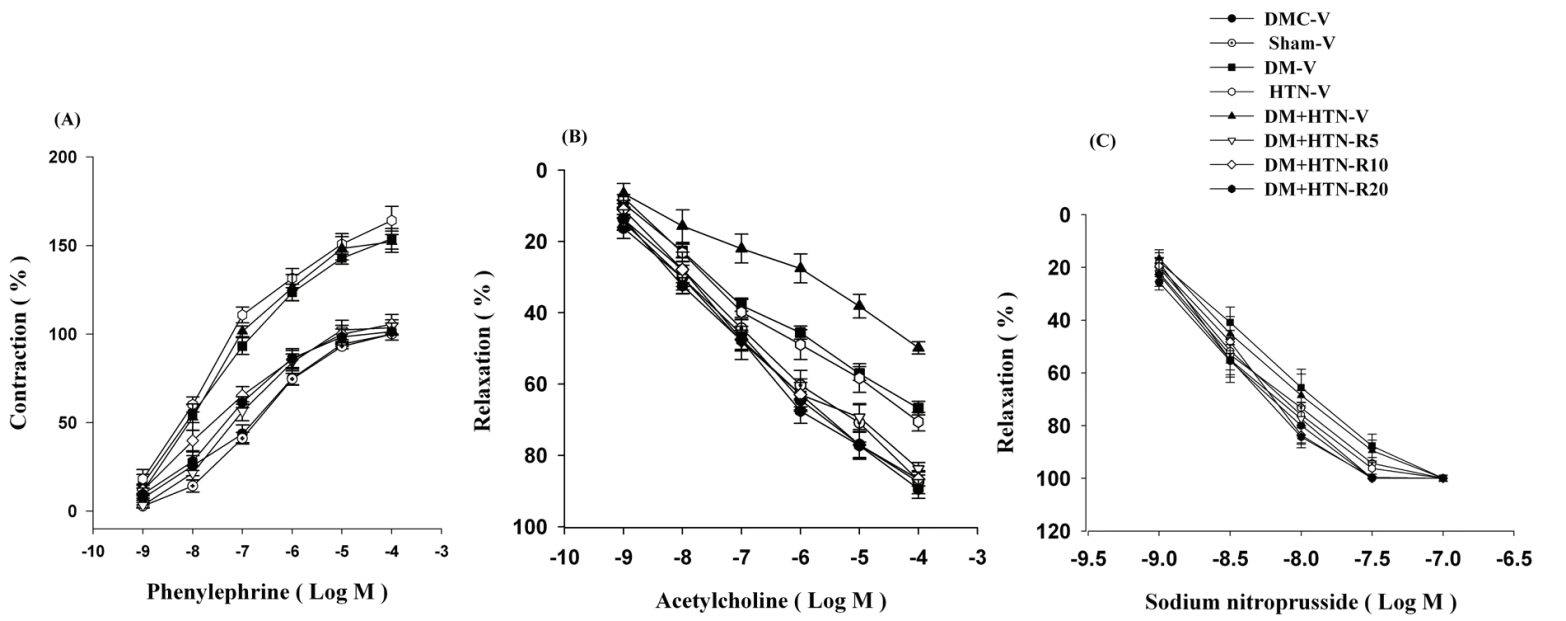
Dose responses from isolated aortic ring studies for all experimental groups after 4 weeks of treatment with vehicle or resveratrol. DMC-V: Diabetic control group receiving vehicle; Sham-V: Sham-operated receiving vehicle; DM-V: Diabetic receiving vehicle; HTN-V: Hypertensive receiving vehicle; DM+HTN-V: Diabetic hypertensive receiving vehicle; DM+HTN-R5: Diabetic hypertensive receiving 5 mg/kg/day resveratrol; DM+HTN-R10: Diabetic hypertensive receiving 10 mg/kg/day resveratrol; DM+HTN-R20: Diabetic hypertensive receiving 20 mg/kg/day resveratrol; Data are mean±SEM and n=8-10 in each group; *Significant difference (P<0.05) from DMC-V or Sham-V; ∆Significant difference (P<0.05) from DM+HTN-V

The E_max_s of Phe concentration-responses in DM-V, HTN-V and DM+HTN-V groups were significantly higher than those of DMC-V and Sham-V groups. The E_max_s of concentration-responses to Phe of DM+HTN-R5, DM+HTN-R10 and DM+HTN-R20 groups were significantly lower than that of the DM+HTN-V group (figure 2A, table 2). The EC_50_ of contraction responses to Phe of HTN-V, DM-V and DM+HTN-V groups were significantly lower than those of the DMC-V or Sham-V groups. The EC_50_s of contraction response to Phe from DM+HTN-R5, DM+HTN-R10 and DM+HTN-R20 groups were significantly higher than that of the DM+HTN-V group (table 2).

**Table 2:** The values of pharmacological variables obtained from isolated aortic ring studies after 4 weeks of treatment with vehicle or resveratrol

	**Phenylephrine**		**Acetylcholine**	
	** E_max_**	** EC_50_**	** E_max_**	** IC_50_**
DM2-C-Veh	100.0±0.0	-6.76±0.08	87.5±3.2	-7.23±0.13
DM2-Veh	153.7±26.8*	-7.04±0.09*	66.7±1.9*	-6.63±0.26*
Sham-Veh	100.0±0.0	-6.77±0.10	87.0±1.5	-7.13±0.13
HTN-Veh	173.8±18.2*	-7.37±0.08*	70.5±2.6*	-6.69±0.28*
DM2+HTN-Veh	165.1±16.0*	-7.29±0.10*	49.8±1.7*	-6.15±0.37*
DM2+HTN-Res5	103.6±9.3^Δ^	-6.10±0.08^Δ^	83.8±1.7^Δ^	-7.07±0.16^Δ^
DM2+HTN-Res10	105.5±13.4^Δ^	-6.91±0.12^Δ^	86.7±2.0^Δ^	-7.16±0.21^Δ^
DM2+HTN-Res20	101.4±8.8^Δ^	-6.70±0.15^Δ^	87.5±3.2^Δ^	-7.3±0.12**^Δ^**

DMC-V: Diabetic control group receiving vehicle; Sham-V: Sham-operated receiving vehicle; DM-V: Diabetic receiving vehicle; HTN-V: Hypertensive receiving vehicle; DM+HTN-V: Diabetic hypertensive receiving vehicle; DM+HTN-R5: Diabetic hypertensive receiving 5 mg/kg/day resveratrol; DM+HTN-R10: Diabetic hypertensive receiving 10 mg/kg/day resveratrol; DM+HTN-R20: Diabetic hypertensive receiving 20 mg/kg/day resveratrol; E_max_: Maximal response; EC_50_: Effective concentration 50; IC_50_: Inhibitory concentration 50; EC_50_ and IC_50_ are presented as antilog of the concentrations; Data are mean±SEM and n=8-10 each; *Significant difference (P<0.05) with DMC-V or Sham-V; ^∆^Significant difference (P<0.05) with DM+HTN-V

The E_max_sof ACh concentration-responses of DM-V, HTN-V and DM+HTN-V groups were significantly lower than those of DMC-V or Sham-V groups. The E_max_s of ACh concentration-responses of DM+HTN-R5, DM+HTN-R10 and DM+HTN-R20 groups were significantly higher than those of the DM+HTN-V group (figure 2B, table 2). The IC_50_s of relaxation response to ACh of HTN-V, DM-V and DM+HTN-V groups were significantly higher than those of the DMC-V and Sham-V groups. The IC_50_s of concentration-response to ACh of DM+HTN-R5, DM+HTN-R10 and DM+HTN-R20 groups were significantly lower than that of the DM+HTN-V group (figure 2B, table 2). There was no significant difference in IC_50_ or E_max_sof SNP concentration-responses in all groups (figure 2C).

## Discussion

The main objective of the present study was to examine the resveratrol’s antihypertensive and antidiabetic effects and their possible mechanisms in rats with simultaneous type 2 diabetes mellitus and renal hypertension. The study showed that resveratrol had antihypertensive, antidiabetic and antioxidative stress activities, and prevented the impairment of endothelial release of NO.

The present study showed that administration of NA and STZ resulted in type 2 diabetes, which its blood glucose was in the range reported by us^19^ and others.^20^ This study also shows that, similar to our previous studies^19^ and those of others,^8^ the present model was associated with increased oxidative stress.^15^ Our findings show that placement of plexiglass clips on the left renal arteries led to renal hypertension, in which the increase in blood pressure was comparable to those obtained in our earlier studies^21^^,^^22^ and those of others.^23^ The findings also shows that administration of NA and STZ and placement of plexiglass clips resulted in a model of simultaneous type 2 diabetes and renal hypertension, which similar to our previous studies^22^ was associated with the increased blood glucose, hypertension, impaired cardiac function, impaired endothelial function, and increased oxidative stress. Possible mechanisms of increased blood pressure, blood glucose, and cardiac dysfunction in the present models of type 2 diabetes, renal hypertension, and simultaneous type 2 diabetes and renal hypertension were discussed in our earlier publications.^17^^,^^22^

The present study represents the first to report that resveratrol, at all examined doses, was antihypertensive in a rat model of simultaneous type 2 diabetes and renal hypertension. This finding is in agreement with previous studies demonstrating that resveratrol had antihypertensive effects in DOCA-salt hypertensive rats,^3^ spontaneously hypertensive rats,^2^ experimental model of malignant hypertension^24^ and two-kidney, one-clip hypertensive rats.^4^

The mechanism of antihypertensive activity of resveratrol is not exactly known, but a previous report has attributed that to increased release of endothelial NO rats.^25^ In order to investigate the mechanism of resveratrol’s antihypertensive effects, we examined whether it might be related to the status endothelial-derived NO release. Similar to experimental diabetes^26^ or experimental hypertension,^3^ our model of simultaneous type 2 diabetes and renal hypertension was associated with impaired release of endothelial NO. The Impaired relaxation response to ACh is an indication of reduced release of nitric oxide.^17^^,^^27^ Our findings show that E_max_ and EC_50_ of relaxation response to ACh in aortic rings from rats with simultaneous type 2 diabetes and renal hypertension were significantly lower and higher than those of the control group, respectively. These findings indicate that the model was associated with impaired release of nitric oxide. However, resveratrol restored the reduced E_max_ and increased IC_50_ of ACh relaxation response, which indicate that it prevented the impairment of endothelial release of NO. Enhancement of endothelial-derived NO activity underlies the antihypertensive activity of a number of other drugs such as enalapril and quinapril,^28^ nebivolol and carvedilol,^29^ losartan^30^ or extracts of herbs such as garlic^31^ and crataegus.^32^ The resveratrol’s preservation of endothelium-derived NO release is in agreement with previous studies demonstrating that it increased the release of endothelial NO in spontaneously hypertensive,^2^ obese Zucker,^33^ two-kidney, one-clip hypertensive,^4^ fructose-fed,^9^ and healthy^34^ rats.

We also examined whether resveratrol’s sympatholytic activity might be involved in its antihypertensive effects. Our findings show that resveratrol significantly prevented the increase of heart rate in rats with simultaneous type 2 diabetes and renal hypertension. It also prevented the increase in contraction response to Phe in aortic rings from such rats. Both such effects may have contributed to antihypertensive activity of resveratrol. The prevention of heart rate increase and attenuation of contractile response to Phe may be indicative of resveratrol’s beta and alpha antagonism, respectively. Alternatively, resveratrol may have acted somewhere upstream to the alpha and beta receptors, and have caused the attenuation of sympathetic nervous system activity, which has been reported to be elevated in renal hypertension.^4^

The increased oxidative stress has been reported to be involved in the development of hypertension by causing up-regulation of angiotensin II signaling,^35^ decreasing NO bioavailability,^34^ dysfunction of endothelial NO synthase,^9^ and reduction of the levels of reactive oxygen species scavenger.^34^ We therefore, measured the concentration of serum markers of oxidative stress. The findings of the present study indicate that the model of simultaneous type 2 diabetes and renal hypertension was associated with the increased oxidative stress, and treatment with resveratrol prevented the increase of oxidative stress, characterized by decreased serum levels of MDA, and increased serum levels of SOD. This finding is similar to those of previous studies showing that resveratrol decreased oxidative stress in stroke prone spontaneously hypertensive,^36^ two-kidney, one clip hypertensive,^4^ partially nephrectomized^37^ and fructose-fed^9^ rats. Given the role of increased oxidative stress in the development of hypertension, it might be possible to speculate that, by virtue of antioxidative stress activities, resveratrol might have offset one or more of above-mentioned mechanisms, and thereby prevented the increase of blood pressure. However, a recent publication suggests that resveratrol, by virtue of its antioxidative effects, increased phosphorylation of adenosine-monophosphate-activated protein kinase, and thereby reduced blood pressure.^9^

The present study shows that resveratrol did decrease the blood glucose in rats with simultaneous type 2 diabetes and renal hypertension. This finding is similar to those of previous studies showing that resveratrol decreased blood glucose in streptozotocin-induced,^13^ and streptozotocin and nicotinamide-induced^15^ diabetes. Several mechanisms such as preservation of beta cells,^12^ increased release of insulin,^13^ enhancement of insulin sensitivity,^14^ increasing insulin receptor substrate-1 and glucose transporter 1 and 4,^38^ and decrease of oxidative stress^15^ have been proposed to mediate resveratrol’s antidiabetic effect.

In order to investigate whether antidiabetic effect of resveratrol may be attributed to antioxidative stress properties, serum concentrations of MDA and SOD were measured. Treatment of rats with simultaneous type 2 diabetes and renal hypertension was associated with reduced oxidative stress activities. Such findings are in agreement with previous studies showing that resveratrol decreased oxidative stress in type 2 diabetic patients,^11^ streptozotocin-induced diabetes,^13^ streptozotocin and nicotinamide-induced diabetes,^15^ and fructose-fed rat.^9^ Therefore, it is appropriate to suggest that by reducing the oxidative stress, resveratrol may have reduced blood glucose in the present study.

Diabetes mellitus and hypertension often occur simultaneously in human. Data from Farmingham study indicate that at the time of diabetes diagnosis, 58% of patients had hypertension.^39^ Simultaneous occurrence of these diseases is associated with a higher cardiovascular risk and mortality in human.^40^ Given the grave consequence of simultaneous occurrence of the two diseases, the use of a single remedy that is effective for both diseases are of vital importance. Our findings show that resveratrol did have both antihypertensive and antidiabetic activities. Therefore, our findings may open a further horizon for resveratrol’s research, and its future use as a supplement or a drug in situations that the two diseases occur concurrently.

## Conclusion

The findings of the present study indicate that resveratrol has antihypertensive and antidiabetic effects in rats with simultaneous renal hypertension and type 2 diabetes. Resveratrol effects might be partly attributed to relate to restoration of endothelial NO release, a sympatholytic or α and β receptor blocking activity, and antioxidative stress activity. 
